# A first approach to evaluate the cell dose in highly porous scaffolds by using a nondestructive metabolic method

**DOI:** 10.4155/fso.15.58

**Published:** 2015-11-01

**Authors:** Carla Divieto, Maria Paola Sassi

**Affiliations:** 1 INRIM – Istituto Nazionale di Ricerca Metrologica, Strada delle Cacce, 91 – 10135, Torino, Italy

**Keywords:** Biocoral^®^, cell dose, cell number, cell proliferation, hMSCs, nondestructive methods, porous scaffolds

## Abstract

**Background:**

In cell-based therapies, *in vitro* studies on biomimetic cell–scaffold constructs can facilitate the determination of the cell dose, a key factor in guaranteeing the effectiveness of the treatment. However, highly porous scaffolds do not allow a nondestructive evaluation of the cell number. Our objective was to develop a nondestructive method for human mesenchymal stem cells dose evaluation in a highly porous scaffold for bone regeneration.

**Materials & measurement method:**

Proliferation trend of human mesenchymal stem cells on Biocoral^®^ scaffolds was measured by a resazurin-based assay here optimized for 3D cultures. The method allows to noninvasively follow the cell proliferation on biocorals over 3 weeks with very high reproducibility.

**Conclusion:**

This reliable method could be a powerful tool in cell-based therapies for cell dose determination.

## Abstract (lay abstract)

Stem cells regenerate damaged tissues when transplanted into the patient within matrices mimicking the tissues architecture and mechanical properties. Cell number needs to be appropriate to allow the cell survival in the new environment and to stimulate the cell differentiation into the new tissue. *In vitro* experiments give important hints to determine the appropriate number to transplant in the patient: in this study cells are grown on highly porous matrices for bone regeneration and their number is monitored over time by a method which does not perturb the system and which was here optimized and evaluated as highly reliable.

**Figure 1.**
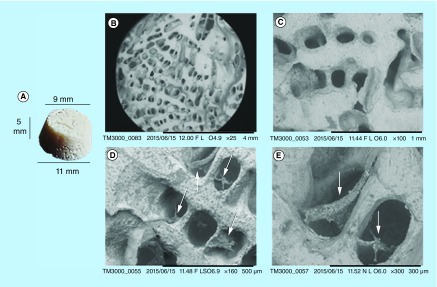
**Biocoral scaffolds images.** **(A)** Biocorals have been acquired with digital camera, and **(B–E)** scanning electron microscopy. **(B & C)** show a scaffold before seeding: in **(B)** the scaffold surface where cells are usually seeded (scale bar = 4 mm) and in **(C)** the scaffold internal pores, after scaffold mechanical fragmentation(scale bar = 1 mm). **(D & E)** show a scaffold after cell seeding andcell fixation: in **(D)** fixed cells (arrows) occupied some pores (bar =500 μm) and in **(E)** at higher magnification two cells in two different pores (arrows; scale bar = 300 μm).

**Figure 2.**
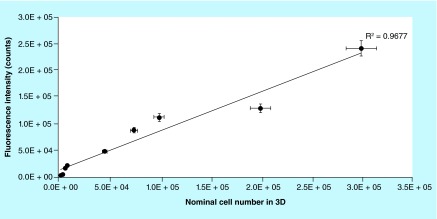
**Calibration curve for 3D cell culture on Biocoral^®^.** Fluorescence intensity of resazurin metabolized by cells on biocorals after 2 h incubation. The correlation between the fluorescence intensity and the nominal cell number, was found linear in the range between 5 × 10^3^ and 3.0 × 10^5^ cells. The correlation coefficient was 0.967. Error bars: horizontal bars are the uncertainty if the manual counting estimated as 5%; vertical bars are the uncertainty of the fluorescence measurements estimated as 5.9%.

**Figure 3.**
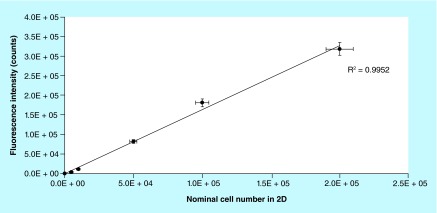
**Calibration curve for 2D cell culture.** Cells were seeded in six-well plate in the range of 1.0 × 10^3^ and 2.5 × 10^5^ per well. Resazurin was incubated with cells for 2 h. The correlation coefficient was 0.995. Error bars: horizontal bars are the uncertainty if the manual counting estimated as 5%; vertical bars are the uncertainty of the fluorescence measurements (estimated as 5%), n = 3.

**Figure 4.**
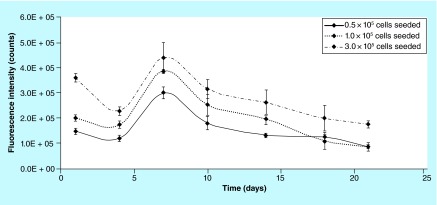
**Experimental reproducibility: fluorescence intensity on biocorals seeded with 0.5, 1.0 and 3.0 × 10^5^ cells.** Mean fluorescence intensity from human mesenchymal stem cells at P7 seeded at three nominal cell number (0.5 and 1.0 and 3.0 × 10^5^ cells) on biocorals (n = 3 for 0.5 and 1.0 × 10^5^ cells; n = 6 for 3.0 × 10^5^ cells) and measured over time at days 1, 4, 7, 10, 14, 18, 21. Error bars represent the uncertainty of the measurement calculated quadratically composing the experimental repeatability and the experimental reproducibility.

**Figure 5.**
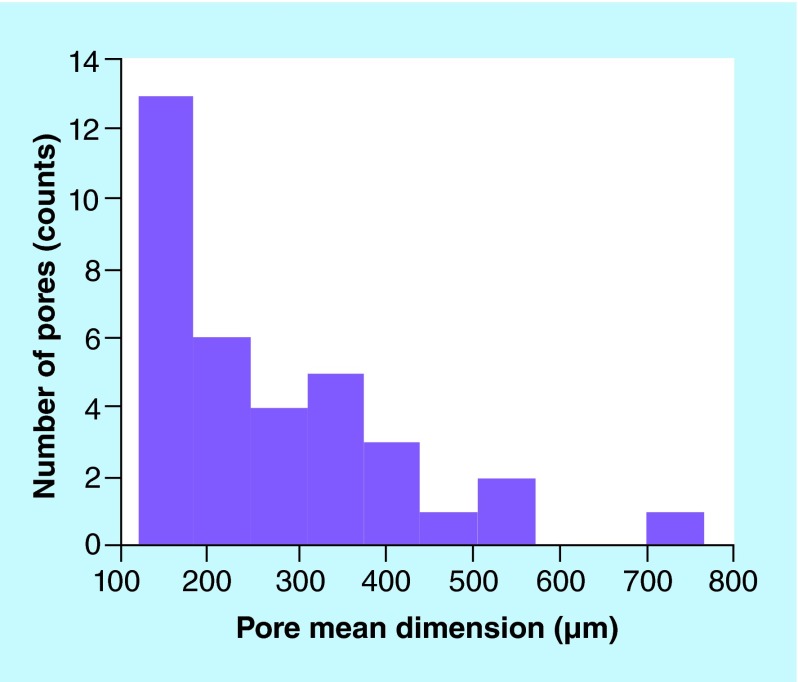
**Pore distribution of a representative portion of Biocoral^®^ scaffolds.** The pore size distribution ranges from 100 to 700 μm.

**Figure 6.**
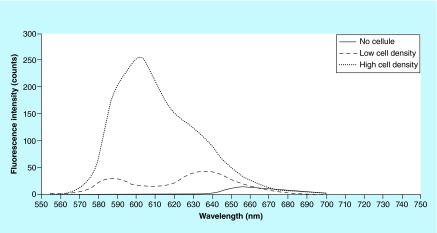
**Resazurin and resorufin emission spectra.** A low number and a high number of cells after 2 h incubation metabolize differently a resazurin 44 μl solution. The excitation was at 540 nm and the maximum emission (peaks) was found around 635 nm for resazurin metabolized by low number of cells (i.e., low concentrated resorufin) and 600 nm for resazurin metabolized by high number of cells (i.e., high concentrated resorufin).

**Figure 7.**
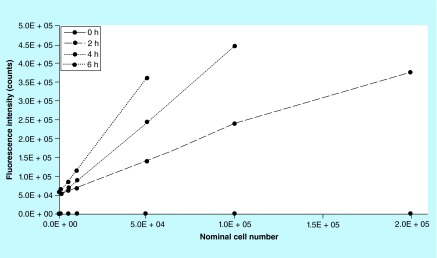
**Incubation time is cell number dependent.** Cells seeded at higher density need a shorter incubation time to avoid off-scales. Points on curves at 4 and 6 h were off-scale after 5.0 × 10^4^ and 1.0 × 10^5^, respectively (missing points on curves). The optimal incubation time was found to be 2 h allowing to measure intensity on the entire range of cell number with no need for dilution.

**Figure 8.**
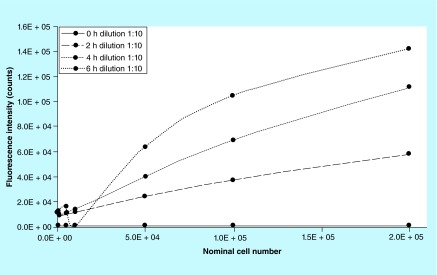
**Effect of dilution.** Dilution 1:9 of the same metabolized resazurin analyzed and shown in Figure 4 are represented here.

**Figure 9.**
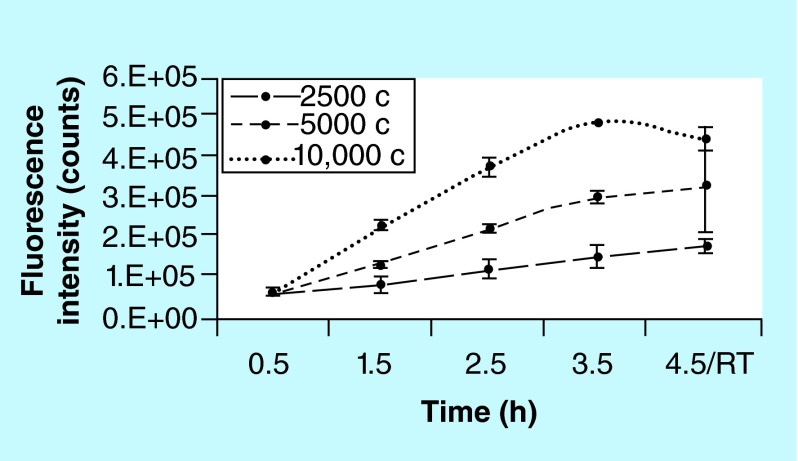
**Incubation time and temperature effect.** The fluorescence measurement should be done as soon as the cells, or the metabolized resazurin, are taken from the incubator. The temperature decrease modifies the fluorescence intensity presumably due to the temperature-dependent pH decrease in absence of CO_2_ or other pH buffers. Error bars represent the standard deviation of three samples (n = 3).

**Figure 10.**
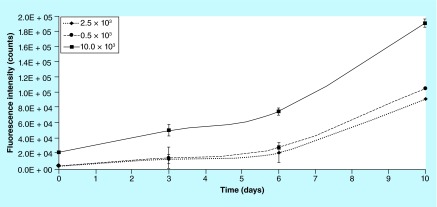
**Sensitivity of the method: fluorescence intensity on biocorals seeded with 2.5, 5.0 and 10.0 × 10^3^ cells.** Error bars (n = 3) represent the uncertainty of the measurement, calculated quadratically composing the fluorescence intensity measurement uncertainty and the experimental repeatability.

**Figure 11.**
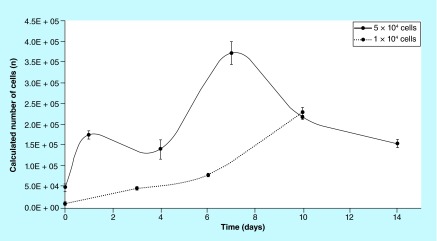
**Cell proliferation on Biocoral^®^.** Proliferation curves on Biocorals expressed as number of cells (n) versus time (days in culture). Scaffolds seeded with 1.0 × 10^4^ nominal human mesenchymal stem cells and scaffolds seeded with 5.0 × 10^4^ nominal human mesenchymal stem cells. Error bars are the standard deviation among three scaffolds (n = 3).

**Figure 12.**
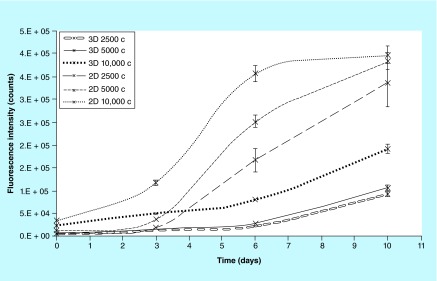
**2D and 3D cell cultures proliferation trend.** Fluorescence intensity emitted by cells as function of the days in culture. In 2D cell cultures at high density the proliferation trend showed the typical lag and log phases and a final plateau, the medium and low densities showed a lag and log phases both supposed being close to reach the plateau. In 3D cell cultures the trend is very similar in all three initial densities, however the lag phase is much longer and the log phase just starts at day 6. Error bars represent the uncertainty of the measurement as described in the Materials & measurement method section ‘Experimental repeatability and reproducibility on 3D cell cultures’.

**Figure 13.**
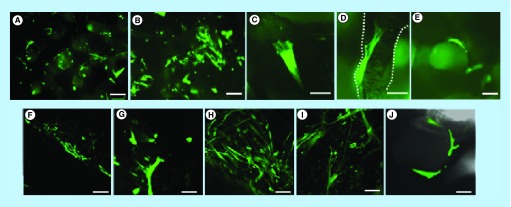
**Cell viability assay: cell colonization of external and internal surfaces.** Fluorescence microscopy and confocal microscopy images of human mesenchymal stem cells on Biocorals^®^. In green are shown human mesenchymal stem cells after 7 and 21 days of 3D cell culture at 5×, 10×, 20× and 40× magnification. In **(A–E)** cells were imaged after 7 days in 3D culture by fluorescence microscopy: in **(A)** and **(B)** cells within the Biocoral^®^ pores appear black with rounded shape in **(A)**, bar = 150 μm; in **(C)** and **(D)** details of two single cells adherent to the scaffold structure, bar = 50 μm; in **(E)** a cell adherent to the wall of a single pore bar = 100 μm. In **(F–J)** cells were imaged after 21 days in 3D culture by confocal microscopy: in **(F)** cells on the external surface delineating the scaffold boundaries, bar = 150 μm; in **(G)** cells within the scaffold, bar = 50 μm; in **(H)** and **(I)** cells on the surface, bar = 100 μm; in **(J)** it is visible one pore containing three cells bar = 50 μm.

Stem cell-based clinical applications in the fields of cell therapy, regenerative medicine and tissue engineering, require that a certain number of cells is delivered for implantation into the patient to actively participate to the regenerative process of damaged or injured tissues [1,2]. Typically human mesenchymal stem cells (hMSCs) are isolated after bone marrow aspiration, cultured *in vitro* and reimplanted alone by injection or in combination with 3D matrices (or scaffold) by insertion of the 3D cell–scaffold complex [3–5]. hMSCs are primary cells with the capability to self-renewal and to differentiate into osteogenic, adipogenic and chondrogenic lineages [4,5]. By combining hMSCs with 3D scaffolds made of different biomaterials, tissue engineering is able to provide tissue-specific *in vitro* 3D models which mimic the *in vivo* environment for studies in the fields of regenerative medicine, human diseases, new therapies development and diagnostics [6,7]. In clinical applications, MSCs, in combination with other factors (e.g., growth factors, mechanical and chemical properties of the biomaterial itself), will both stimulate and initiate the tissue repair process by differentiating into specific cell lineage or by secreting factors which in turn stimulate other specialized cells to repair the damaged tissue [3,8]. In this complex process MSCs proliferation and differentiation coexist in a dynamic equilibrium [9]. The appropriate cell number, the so-called cell dose, of MSCs is an important factor to be monitored in order to obtain the desired final result: it is crucial for the cell differentiation process *in vitro* for research studies because the cell number will affect the cell confluency (cell number per surface area) and the cell confluence strongly influences the starting of the cell differentiation process [10]; it is strongly relevant to obtain the scaffold colonization preimplantation if considering that the same cell number in 2D and 3D give different proliferation rate [11]; it is one of the main variables affecting the cell survival in *in vivo* cell–scaffold implantation, together with biological state of the cells (resting, proliferation, differentiation) and with the oxygen and nutrient concentration and diffusion [3,12]. In addition, the cell dose estimation allows to correlate the patient response to the treatment and, consequently, to choose the most appropriate initial cell number to reach the best cell therapy final result. The possibility to monitor continuously over time and in real time the same 3D model, without modifying or perturbing the system, is of great importance to investigate cellular behavior and functions and to translate the data obtained *in vitro* into meaningful prediction of the *in vivo* effects. Biocoral^®^ (Inoteb, LeGuernol, Saint-Gonnery, France) scaffold is a 3D natural coralline scaffold used as good candidate biomaterial to support the formation of new bone as demonstrated by *in vitro* and *in vivo* studies [13,14]. Preclinical and clinical trials evaluated and demonstrated the Biocoral biocompatibility with human cells and the ability to support cell growth and an osteogenic differentiation [15], the osteointegration and the resorption of Biocoral in human bone [16] and the ability to convey cytokines [17]. Clinical applications of biocorals are mainly active in the field of dentistry and periodontal surgery [18].

However, even if Biocoral scaffolds offer interesting features for bone regeneration, the evaluation of the cell dose effect in a noninvasive manner (i.e., without fixing, slicing or staining the cell–scaffold construct) is still a challenge. To investigate the cell dose, cell viability and proliferation are fundamental phenomena to be monitored. By quantitatively monitoring cell viability and proliferation dynamics it is possible to get information on effectiveness of a cell–scaffold 3D complex. In addition, viability and proliferation are parameters giving insights and allowing predictions about other cellular processes such as cell differentiation. Likely due to their highly porosity Biocoral are not easily permissive to traditional methods for cell number and cell proliferation assessment (e.g., manual cell counting after trypsinization and MTT assay, among others) and because of their opacity do not allow traditional imaging by contrast phase optical microscopy and, moreover, fluorescence imaging of labeled cells is only possible on the scaffolds surface. It is necessary to fix, slice or stain the sample [19] and this prevents any real time, or a time-course nondestructive study. Several studies investigated the possibility for noninvasive or minimally invasive monitoring over time cellular functions and behavior [20–22]. However, to our knowledge standardization and characterization of a nondestructive method for monitoring over long period of time 3D cell cultures proliferation and behavior is still missing.

This work aims to propose a nondestructive method to estimate the cell dose and to evaluate the cell proliferation in 3D cell cultures on highly porous scaffolds. The feasibility and reliability of the method as a cell growth indicator in coralline culture systems has been evaluated in long-term experiments on 21 biocorals. The method is based on the cellular metabolization of the resazurin dye. The resazurin assay is an indirect, fluorimetric, metabolic assay to estimate the number of living cells in *in vitro* systems by measuring a fluorescence intensity which is proportional to the number of metabolically active living cells [23–25]. Only living cells can perform this reaction because nonviable cells rapidly lose metabolic capacity and do not generate any fluorescent signal [25]. Resazurin has been extensively used in traditional 2D cell cultures to assess the cell cytotoxicity and proliferation [24–27], and only recently it has been used on scaffolds to assess the cytotoxicity of the scaffold itself [28–31]. However, resazurin-based methods for cell proliferation assessment have never been well characterized for 3D cell cultures in term of linearity, sensitivity, reproducibility and reliability of the method, and has never been used on coralline and highly porous scaffolds, such as the Biocoral.

## Materials & measurement method

### Biocoral scaffolds

Biocoral is a highly porous, rigid and inert scaffold having a chemical composition very similar to that of human bone (98% of calcium carbonate, 1% of strontium able to promote the mineralization process and other trace elements). Its porosity varies from 20 to 50% of the total volume and its internal architecture has highly interconnected pores of 150–500 μm in diameter. Biocorals used in this study, have truncated cone shape with 9 mm in minor diameter, 11 mm in major diameter and 5 mm in height as shown in Figure 1A (Samsung L201 digital camera). A scanning electron microscope (TM 3000 Hitachi, Tokyo, Japan) at 25×, 100×, 150× and 300× magnification was employed to image the surface and the internal structure of three different Biocoral. The internal structure was imaged after mechanical fragmentation of biocorals. The Biocoral pore dimension distribution was obtained by imaging a representative number of pores with an Axio Observer Z1 optical microscope (Zeiss, Göttingen, Germany) with 20× magnification. A calibrated XY linescale was used to obtain the pixel dimension. Mean diameter of pores (counts = 40) was measured by comparing the length of the segment diameter of pores with the dimension of pixels.

### Mesenchymal stem cells 3D cultures on Biocoral

Bone marrow (BM) derived hMSCs were purchased from Lonza Group Ltd (Basel, Switzerland). BM-hMSCs were maintained as adherent cultures in the cell growth medium prepared with alpha modified Minimum Essential Medium (Lonza, Wokingham, UK) (αMEM) supplemented with 10% v/v FBS (fetal bovine serum, Lonza, Wokingham, UK), 2 mM l-glutamine, 100 IU/ml penicillin and 100 mg/ml streptomycin (Lonza, Wokingham, UK). Cells were grown in monolayers in Petri dishes at 37°C in 5% CO_2_ and 95% humidity. Fresh medium was replaced every 3–4 days until cells reached about the 80% of confluence.

hMSCs at P7 were washed once with 1× Phosphate Buffer Saline (PBS), detached with 0.25% Trypsin – 0.53 mM EDTA solution, counted by using a hemocytometer (i.e., Neubauer chamber) and resuspended in 100 μl cell growth medium (prepared as described above) at nine different concentrations (nominal cells/100 μl): 2.5 × 10^3^, 5.0 × 10^3^, 7.5 × 10^3^, 10.0 × 10^3^, 0.5 × 10^5^, 0.75 × 10^5^, 1.0 × 10^5^, 2.0 × 10^5^ and 3.0 × 10^5^ (n = 3 for each cell concentration). Each 100 μl drop of cells suspension was deposited on the upper surface of a Biocoral, previously placed into a well of a 24-well plate. Scaffolds with cells were incubated at 37°C and 5% CO_2_ for 1 h to let the drop be absorbed and to allow the cells to adhere on and within the scaffold. After 1 h, 1 ml of cell growth medium was added to each well until completely covering the scaffold. Cells were cultured statically on 21 weeks, the growth medium was refreshed every 2–3 days and the cell proliferation assay was performed at several time points for linearity assessment of the method and for cell proliferation analysis over time. A sample of Biocoral without cells treated in the same conditions was used as negative control. No osteogenic medium nor other osteoinductive factors were added.

### Resazurin assay

The CellTiter-Blue^®^ (CTB) cell viability assay (Promega Corporation, WI, USA) was used as resazurin-based method. The CTB reagent is a buffered solution containing highly purified resazurin. This assay is based on the reduction of resazurin to resorufin: resazurin is a redox dye with a slight intrinsic fluorescence, dark blue in color, while resorufin is a highly fluorescent molecule, pink in color, with excitation wavelength of 579 nm and emission maximum at the wavelength of 584 nm [23]. The resazurin molecule can penetrate the cells by passing the cell membrane and into the cytoplasm is reduced by cytosolic, microsomal and mitochondrial redox enzymes producing the fluorescent resorufin. Resorufin diffuses out of cells back to the culture medium which alone does not reduce resazurin [23,32]. A 10% of CTB in fresh αMEM was used as resazurin working solution. CTB at 10% correspond to 44 μM as demonstrated by Xiao *et al*. [30]. Fluorescence intensity of resazurin solution was measured with the GloMax^®^-Multi Microplate Multimode Reader (Promega Corporation) by using the optical kit of filters for resorufin, typically used for CTB assay having a preset excitation peak wavelength at 525 nm and an emission wavelength in the range of 580–640 nm.

In addition, to characterize the fluorescence spectra, fluorescence measurements of solution of nonmetabolized resazurin 44 μM (control sample) and resazurin metabolized by a low and a high number of cells (resorufin at two different concentration) in cell culture medium were carried out using a F-4500 fluorescence spectrophotometer (Hitachi Co., Ltd, Tokyo, Japan). For each of the three different solutions, a volume of 700 μl was scanned at several excitation wavelengths in the range of 500–600 nm and the fluorescence emission spectrum was investigated in the range of 550–700 nm at 60 nm/min scan speed.

### Instrumental repeatability & reproducibility

The instrument GloMax-Multi Microplate Multimode Reader (Promega Corporation) has been well characterized in term of fluorescence measurement repeatability and reproducibility, calculated as showed in an our previous study [33]. Briefly, the repeatability of the fluorescence measurement has been evaluated by repeating the measurement ten-times for each single well of a 96-well plate. The standard deviation of repeated measurements was calculated and was found lower than 0.5%. The reproducibility evaluated among the 96 wells on the same plate due to the position of the well within the plate has been found lower than 1%. In addition, a very important parameter influencing the measurement has been found in the reading volume of resazurin: the level of the liquid filling the well, in the 96-well plate, influences the fluorescence intensity. The fluorescence intensity was highest in the range around 100 μl. Measurements were repeated in triplicate in order to identify outliers.

### Resazurin assay for 3D cell cultures on Biocoral

#### Optimization experiments

In order to establish a protocol and to optimize the resazurin-based method for 3D cell cultures, a series of experiments were performed on Biocoral to identify the better experimental conditions in terms of resazurin concentration, incubation time and cell growth medium composition.

To evaluate any possible interference between auto-fluorescence of cell culture media and fluorescence intensity of resazurin, two culture media, alpha Modification of Eagle's Medium (αMEM) and Dulbecco's Modified Eagle Medium (DMEM), both commonly used for BM-hMSCs, were tested: αMEM with phenol red, with FBS 1% and 10% (V/V) and DMEM without phenol-red, with FBS 1% and 10% (V/V).

Two resazurin concentrations in the selected cell culture medium were tested: 44 μM and 88 μM, corresponding to 10 and 20% respectively (V/V) of the commercial product AlamarBlue [30].

Incubation times of 0.5, 1, 1.5, 2, 2.5, 3, 4 and 6 h were tested to find the most suitable time able to allow analysis in the entire range of number of cells we want to test.

Results coming from optimization preliminary experiments were used to perform the proliferation assays on Biocoral on 21 days (described in the Results section, ‘Experimental repeatability and reproducibility on 3D cell cultures’).

#### Calibration curve & sensitivity of the method

To define the calibration curve and assess the linearity of the method in 3D, a serial number of cells (Figure 2) was seeded on biocorals and fluorescence was measured after 2 h incubation with resazurin 44 μM. The curve has been calculated by the regression analysis of fluorescence intensity experimental data and the nominal value of cells in culture. The nominal value of cells in culture is measured by detaching and counting the cell by means of a hemocytometer, the Neubauer chamber. A sample without cells was used as negative control and its fluorescence intensity values were subtracted as blank. The calibration curve was determined also for 2D cell cultures in six-well plates to compare the linearity in 2D and 3D cell cultures (Figure 3).

#### Experimental repeatability & reproducibility on 3D cell cultures

Experimental repeatability and the experimental reproducibility for fluorescence intensity were calculated to evaluate the combined standard measurement uncertainty, as extensively described in [33] according to [34], and as here briefly described in Equation 1:
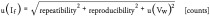



where *I_f_* is the fluorescence intensity and *V_w_* is the reading volume in the well of the 96-well plate. For each scaffold the repeatability is the mean of standard deviation of the three repeated measurements of the same sample and the reproducibility is calculated as the standard deviation the three samples seeded at the same cell density. For each scaffold the reading volume is 100 μl with an uncertainty due to the pipetting error set as 1%.

Error bars on cell nominal number were given by the variability of cell counting by Neubauer chamber, calculated on repeated measurements as 5% in our previous work.

hMSCs were seeded in triplicate at three nominal cell density (0.5, 1.0 and 3.0 × 10^5^ nominal cells/Biocoral) on nine biocorals in the same day (day 0) and cultured for 21 days. At days 1, 4, 7, 10, 14, 18 and 21 biocorals were tested for cell proliferation with the resazurin-based method and fluorescence intensity was measured (Figure 4). A Biocoral without cells was used as negative control for fluorescence intensity and its resazurin fluorescence value was subtracted as blank.

#### Cell proliferation method on Biocoral

Cell culture on Biocoral was tested for cell number by resazurin assay at several time points (from day 1 to day 21) in culture. At each time point each Biocoral (including the negative control) was transferred into a new well and resuspended in 1 ml of a 44 μM resazurin solution in cell culture medium and incubated at 37°C and with 5% CO_2_ on a rocker for 2 h. Metabolized resazurin was then collected in microcentrifuge tubes. Scaffolds were washed twice with PBS and 1 ml of fresh cell growth medium was added to each scaffold. A volume of 100 μl of metabolized resazurin from each scaffold was transferred in triplicate into a 96 well-microplates and fluorescence intensity was measured with the GloMax microplate reader by using the optical kit of filters for the CTB assay having a preset excitation peak wavelength at 525 nm and an emission wavelength in the range of 580–640 nm.

### Cell viability test

Cell viability was evaluated by Calcein AM staining (Invitrogen – Thermo Fisher Scientific Corporation, MA, USA) at days 7 and 21 after resazurin assay was performed. The Calcein AM molecule is a cell-permeant dye, able to enter the cell and only live cells are metabolized by cytoplasmic intracellular enzymes (esterases). By the hydrolysis of the acetoxymethyl ester (AM) group, enzymes modify the chemical structure of the molecule that is converted from a nonfluorescent calcein into a green-fluorescent calcein. The green fluorescent calcein is retained only within viable cells, producing an intense uniform green fluorescence with excitation wavelength at 495 nm and emission at 515 nm. Calcein AM was added in each well containing the scaffolds in 1 ml of cell culture medium to a final concentration of 5 μM and incubated for 1 h at 37°C. Scaffolds were then washed with PBS and fresh medium was added before cell live imaging. An Axio Observer Z1 optical microscope (Zeiss, Göttingen, Germany) with a mercury vapor lamp for fluorescence excitation, at 5×, 10× and 20× magnification and a laser scanning confocal microscope (LSCM, Zeiss) at 5×, 20× and 40× magnification were used to assess the cell viability.

### Comparison of the method between 2D & 3D cell cultures

We compared the method for cell proliferation measurement in 2D and 3D cell culture by evaluating the fluorescence intensity of the metabolized resazurin on the same number of cells seeded in traditional 24-well plates (2D) and on biocorals (3D). Three cell densities were analyzed in triplicate: 2.5, 5 and 10 × 10^3^ cells per well or per scaffold. Resazurin assay was performed, as described above, 24 h after the seeding: 1 ml of cell growth medium containing resazurin at a final concentration of 44 μM was added to the cells in 2D and 3D cell cultures and incubated for 2 h at 37°C in 5% CO_2_ and 95% humidity.

## Results

### Biocoral structure & pore size distribution

Dimension of a representative Biocoral is shown in Figure 1A. Scanning electron microscopy imaging (Figure 1B–E) showed the porous structure of biocorals and cells colonizing the pores. Seeding surface where cells are deposited and internal pores were imaged (Figure 1B & C). Scaffolds were imaged after cell seeding and cell fixation: cells occupied the pores by anchoring to the pore walls (Figure 1D & E).

A measurement of the pore size by optical microscopy revealed a pore size distribution ranging from 100 and 700 μm (Figure 5), with a modal value of size around 100 μm.

### Resazurin & resorufin fluorescence spectra

We investigated the differences in terms of fluorescence spectrum of the oxidized (nonmetabolized) and the reduced (metabolized by cells) forms of resazurin. The metabolized form was the product of reaction, the so called resorufin, obtained after 2 h incubation in cell growth medium with two different cell number seeded on two different scaffolds, in other words, low cell density and high cell density. The three solutions analyzed by spectrophotometer (F-4500 Hitachi) revealed three different emission spectra (Figure 6). Interestingly, the metabolized forms of resazurin (which are resorufin metabolized by different cell numbers forming presumably a low and high concentrated resorufin, respectively) present emission peaks in different region of the spectrum. In addition, the one metabolized by a low cell density shows two peaks of interest.

### Optimization experiments

#### Cell culture media do not interfere with the fluorescence emission of resazurin/resorufin

Four cell culture condition was tested: αMEM with phenol red, with FBS 1% and 10% (V/V) and DMEM without phenol-red, with FBS 1% and 10% (V/V). In no one of these cases the medium auto-fluorescence interfered with the measurement of fluorescence intensity coming from resazurin metabolized by cells and high linearity was found in all cases: autofluorescence of media and fluorescence of resorufin were several order of magnitude different. For our purposes, αMEM with phenol red and with FBS 10% was taken as the most appropriate medium mixture because it is routinely used to culture hMSCs in our laboratory, consequently cells are not subjected to any medium change during the assay incubation time. Optimization experiments also demonstrated that no significant interference was found between the fluorescence intensity emission of resazurin and the chemical composition of the Biocoral itself without cells (i.e., the control used as blank).

#### Resazurin at 10% avoids off scale values

The concentration of 44 μM (10%) was the most suitable allowing measurement for cells from low to high cell densities (in the range of 1 × 10^3^ and 8 × 10^5^ cells) with no need for diluting the product of reaction due to off scale values.

#### Incubation time of 2 h is optimal for hMSCs on Biocorals

Several incubation times, from 0.5 to 6 h, were tested and 2 h was found being the optimal time for hMSCs on Biocoral to ensure absence of nonspecific interactions potentially effecting the assay outcome and no further reduction of resorufin into a nonfluorescent hydroresorufin, even with high cell densities and to ensure that no off scale occurs. Experiments showed that the incubation time needs to be adjusted depending on the cell number you want to discriminate, so it is cell number-dependent (Figure 7). After 4 h incubation cells seeded at cell density higher than 1.0 × 10^5^ cell per scaffold are not detected due to fluorescence off-scale values and after 6 h the off-scale values occurred after 5.0 × 10^4^ cell per scaffold. By diluting the metabolized resazurin 1:9 with cell culture medium, results gave linear trends of fluorescence intensity except for 6 h that showed a nonlinear trend (Figure 8). In this work, a suitable range of number of cells has been chosen to colonize the Biocoral scaffold with small dimensions (5 × 9 × 11 mm) avoiding off-scale values of fluorescence intensity.

#### The fluorescence intensity is not stable over time & is temperature dependent

In order to identify any possible factor influencing the final results, we estimated if the resazurin fluorescence is stabile after the incubation time and if the storage temperature does give any effect on the final result. We compared in triplicate 3 cell densities in 2D cell culture in 96-well plates. We measured the resazurin fluorescence, kept in contact with cells, at incubation time of 0.5, 1.5, 2.5, 3.5 at 37°C and after 1 h discarded from cells and kept in the dark at room temperature (the 4.5 h point) (Figure 9). The 3 cell density (2.5, 5.0 and 10.0 × 10^3^ cell/well) was chosen to reach three different confluences (respectively, 25, 50 and 100%). A sample without cells was used as negative control and its fluorescence intensity values were subtracted as blank.

Results showed that for 2D cell culture an incubation of 30 min with resazurin is not enough to allow the discrimination of fluorescence from different number of cells; while incubation time from 1.5 to 3.5 h was optimal to discriminate cells in a 24-well plate system from low to high confluence in the considered range. If the temperature decreases for 1 h, the fluorescence decreases (at the higher cell density) or the variability is very large (see the error bars in samples at middle cell density) or slightly increases (at lower cell density).

### Method linearity & sensitivity on 3D cultures

#### Method linearity

Calibration curve, defined to correlate the cells number seeded on Biocoral scaffolds with the fluorescence intensity of the resorufin produced after resazurin reduction, is shown in Figure 2. The regression between the fluorescence intensity and the nominal cell number was found linear in the range between 5 × 10^3^ and 3.0 × 10^5^ cells. The correlation coefficient R^2^ was 0.97. The uncertainty of the nominal seeded cell number was found to be 5% and the uncertainty of fluorescence measurement was found to be 5.9%, calculated as described in the Materials & measurement method section ‘Experimental repeatability and reproducibility on 3D cell cultures’.

Calibration curve was also determined for 2D cell culture in the range between 1.0 × 10^3^ and 2.5 × 10^5^ cells per well to verify the linearity method in 2D traditional cell cultures, to have a positive control for linearity and to compare the proliferation between 2D and 3D (Figure 3).

#### Method sensitivity

The method for cell proliferation measurement in 3D showed that fluorescence increases over time for each scaffold due to cell proliferation, but it was not able to distinguish between 2.5 × 10^3^ and 5.0 × 10^3^ cells seeded on biocorals, as shown in Figure 10. The sensitivity of the method can be considered 5 × 10^3^ cells for hMSCs on Biocorals and is enough for regenerative medicine purposes where cells are usually seeded at higher densities and consequently the proliferation rate is much higher than 5 × 10^3^ cells/day.

### Experimental repeatability & reproducibility on 3D cell cultures

hMSCs at 0.5, 1.0 and 3.0 × 10^5^ nominal cells/Biocoral seeded on 3, 3 and 6 biocorals respectively and cultured for 21 days were analyzed for cell proliferation with the resazurin-based method at days 1, 4, 7, 10, 14, 18 and 21 and fluorescence intensity was measured immediately after 2 h incubation. It is noticeable (Figure 4) that the fluorescence intensity of resazurin metabolized by hMSCs on biocorals followed very similar trends over time demonstrating a high reproducibility of the method. The mean reproducibility among the nine scaffolds was found to be 15%.

Particularly, the mean reproducibility was found to be: 10% between scaffolds seeded with 0.5 × 10^5^ nominal cells, 14.5% between scaffolds seeded with 1.0 × 10^5^ nominal cells and 20.5% for scaffolds seeded with 3.0 × 10^5^ nominal cells. The repeatability calculated as standard deviation of three repeated fluorescence intensity measurement for each scaffold, was found in the range from 0.0 to 5.2%.

### Cell proliferation curves on Biocorals

The calibration curve, shown in Figure 2, was used to compute the cell proliferation curves on biocorals over time obtained by plotting the calculated cell number versus time (days). Cells were seeded at day 0 in the range of 2.5 × 10^3^ and 3.0 × 10^5^ cells/Biocoral and their proliferation was followed over time. Figure 11 reports the difference between two mean proliferation curves obtained after seeding 1.0 × 10^4^ and 5.0 × 10^4^ cells per Biocoral (n = 3 for each cell density). Proliferation in biocorals seeded at 1.0 × 10^4^, is gradual and continuous, whereas the proliferation in biocorals seeded at 5.0 × 10^4^, is fluctuating over time. In addition, after day 7, in scaffolds with the highest number of cells at the seeding, cells gradually decrease their number and at day 10 cells reach the same number (i.e., 225,000 with 5% of variability) even if the scaffolds were seeded with a number of cells five-times higher than the other group.

### Comparison between 2D & 3D cell culture proliferation

The method for cell proliferation measurement was compared between 2D (24-well plates) and 3D (biocorals) cell culture on three cell densities. Results gave a similar trend in all the 2D cell cultures and similar in all the 3D cell cultures (Figure 12). In 2D, cells show a lag phase, a latent period in which cells grow slowly, followed by a high growth rate till reaching the confluence (particularly at higher cell densities). In 3D cells grow slowly till day 6, resulting in a lag phase much longer than the 2D, and only after day 6 they grow slightly more rapidly.

### hMSCs viability assessment

After cell proliferation assessment by resazurin, cell viability was evaluated by Calcein AM fluorescent staining: living cells appeared in green (Figure 13). Fluorescence and confocal microscopy images (Figure 13A–H) showed that cells are alive after resazurin assay at different time points (7 and 21 days), they were spread both on external surface of the scaffold and within the scaffold pores. Images at several magnification show that cells adhered and proliferated within the Biocoral. Single pores containing cells were also imaged (Figure 13E & J).

## Discussion

The main purpose of this study was to develop a noninvasive method for cell number counting within 3D highly porous scaffolds in order to determine the cell dose in regenerative medicine field. Under the assumption that all viable cells are metabolically active cells and that the average of their metabolic activity is stable over time, the method here proposed measures that the cellular metabolic activity carried out only by viable cells and quantifies the number of cells by correlating the fluorescence intensity of metabolized resazurin with the number of viable cells.

We aimed to give an uncertainty evaluation of the measurement taking into account the bias and reproducibility caused, not only the mean and standard deviation of repeated measurement.

By associating a measurement uncertainty to each cell number it is possible to obtain accurate measurements even with a reduced number of measurement. This can contribute to make the nondestructive assay as less invasive as possible for medical and pharmacological research and applications.

We evaluated several influence factors such as the reproducibility of the cell number at the seeding, the pipetting error, the instrumental reproducibility, the dilution factor, the temperature in order to obtain accurate and reliable measurement. The experimental design of this study was based on fundamental preliminary experiments aiming to characterize the instrumental reliability in terms of reproducibility and repeatability and the methodology on 3D cell cultures in terms of incubation time, resazurin concentrations, appropriate cell culture conditions during the measurement, temperature, linearity, sensitivity and cell number ranges. An interesting paper [32] shows how it is important to optimize the method for the cell type and the specific application. Our work considered many aspects also indicated by Rampersad [32], just listed above, and moreover included other important conditions to be tested.

Based on these preliminary results, we evaluated the method experimental reproducibility at different cell initial number seeded on Biocoral. At low initial cell density the method is more reproducible (10% variability) than at high cell density (20% variability). In addition, we considered properties of the scaffold itself that could have an impact of the final result and could influence the feasibility of the method on such 3D cell cultures.

Scaffold porosity is a key property for the new bone growth, as demonstrated by Jones *et al*. and Mygind *et al*. [35,36] and for bone regeneration: we chose to evaluate the reliability of the method on biocorals as representative of the category of highly porous scaffold for bone replacement and regeneration. Highly porous scaffold is not permissive to cell detachment and sample sectioning or fixation is the routinely used method which does not allow multiplex and real time analysis on the same cell population. In addition, Biocoral chemical composition and biomechanical properties are also very important for the final result: they are very similar to those of the human cancellous bone and experimental studies and clinical applications in neurosurgery, orthopedic surgery and dentistry in the last 20 years have shown this material is fully and gradually resorbable, biocompatible and biofunctional [13,15–17], it is well tolerated by both animal and human tissues, its porosity allows a good osteointegration, which lets the new bone to grow, followed by gradual resorption which leaves space for the new bone. Morphology is another scaffold property playing an important role to guarantee the implant success as recently described by Giuliani *et al*. [37]: they show how synchrotron radiation x-ray microtomography (micro-CT) is a relevant technique to characterize in a nondestructive way coralline-derived biomaterials, particularly Biocoral, in terms of microstructure (morphology and internal structure) at high-resolution and in 3D, even when the scaffold is transplanted in patient.

Our results with their associated uncertainty show that when cells are seeded at high densities (number of cells per seeding surface of the scaffold), the cell proliferation fluctuate over time. Whereas cells seeded at lower densities grow gradually but continuously. Our hypothesis is that behavior of cells is strongly influenced from the internal porous structure of the scaffold in combination with the initial cell density, as also found by Grayson *et al*. [10]. When the cell density is high (presumably more than 1.0 × 10^3^ cell/scaffold), cells are able to fill the pores space rapidly. Consequently, they decrease the proliferation rate due to cell contact growth inhibition. After some days, presumably the time necessary to colonize a new pore, cells are again able to proliferate but again, when the cells fill the new pore the proliferation rate decreases, and so on. To support this hypothesis we should consider the relative dimension of cells and pores. We characterized the Biocoral scaffold pore dimension showing a diameter distribution with a peak around 100–150 μm which is compatible with the typical diameter of hMSCs in 2D cell cultures (50–100 μm). Few cells are needed to fill the wall of a single pore as shown in Figure 13E & J and cells also grow within the pore, not only on the wall, as demonstrated by Figure 1E. The colonization of the Biocoral internal structure by cells occurred in about 7 days as shown in Figure 13A & B. After day 7, cells seeded at high number (Figure 11, 5.0 × 10^4^ cells) decrease their proliferation rate, while cells seeded at low number (Figure 11, 1.0 × 10^4^ cells) continue to grow. Interestingly Zhu *et al*. [19] also found a decrease in cell proliferation rate after day 7 but with only 1.6 × 10^4^ cells seeded per scaffold. The spatial distribution of the cells within the scaffold we obtained was similar to that shown by Zhu *et al*. [19] which found that the cells are distributed on the walls of the internal pores of the scaffold. He obtained this spatial distribution of bone marrow-derived mesenchymal stem cells after fixation, sectioning to obtain slides and staining of nuclei with DAPI. We observed the same distribution without fixing and sectioning the samples.

To test the cell viability within the scaffolds, we performed the viability test with the vital staining of calcein AM, demonstrating that cells are still alive after day 7 and 21 (Figure 13A–J) and the decrease in cell proliferation is not due to cell death.

Our hypothesis is that cells are filling all the pores within the Biocoral and consequently are reducing their proliferation rate and metabolism: a kind of contact inhibition in 3D cell culture, very similar to the one occurring in 2D monolayer culture, as also described by Puliafitoa *et al*. and Yu *et al*. [38,39] in epithelial cell models. When cells reduce the proliferation rate due to contact inhibition, if appropriate conditions occurred (such as chemical or mechanical stimulation) they start to differentiate. A secondary hypothesis we made is that after day 10, the balance between proliferation and differentiation shifts toward differentiation due to biochemical and biomechanical stimuli to which the cells are subjected, in other words, chemical composition, stiffness and internal architecture of Biocoral. This hypothesis will be tested by further work on the differentiation induced by Biocoral on the same cell number we tested here.

By comparing our results on the proliferation trend in 2D cell cultures with the one in 3D cell cultures, we obtained an important results: the 3D showed a slower proliferation rate with a longer lag phase, a latent period in which cells grow slowly probably to adapt to and to exploit the new environmental conditions and to synthetize the cellular components necessary for growing. The most likelihood hypothesis is that in 3D cell cultures cells require a longer time to adapt to the new environment and material, to the different nutrient supply regulated by different vapor pressures at the entrance of the pores and channels, etc. In addition, the total (internal and external) growing surface of Biocoral 3D scaffolds is wider than the 24-well plate 2D surface, consequently, even if the initial cell number is identical at the seeding in 2D and 3D, in 3D scaffolds the cell density (cells/area) is lower and cells need more time to reach the appropriate cell confluence necessary to start a growing log phase. This implies that the proliferation rate is slower and cells extend their time to cover the entire available surface. In addition, importantly, we showed that the sensitivity (or detection limit) of the method is higher in 2D (1000 cells) than in 3D (5000 cells). Also Dienstknecht *et al*. [40] showed similar detection limit for resazurin in 2D cell cultures (500 cells). The lower detection limit in 3D cell cultures could be due to the high porosity: pores retain a small amount of resazurin which is not analyzed for fluorescence which is underestimated. This inconvenience could be overcome by prolonging the incubation time to detect even lower number of cells in 3D. The 3D cell cultures proliferation trend and the viability test results, let us to hypothesize that the diffusion of nutrients, oxygen and other active factors through the Biocoral structure was sufficient enough for cell survival for extended periods of time when seeded at low densities (around 1.0 × 10^4^ in Figure 11). However, if we consider the data in Figure 11, it is to be noticed that the conversion of metabolic activity to cell number is not straightforward. It is known that mesenchymal cells can survive quite low levels of oxygenation in Biocoral if an appropriate glucose supply is present [41]. Thus the drop in metabolic activity shown at higher cell densities (5.0 × 10^4^) at day 4 and 10 in Figure 11 does not necessarily mean that less cells are present, the metabolic activity could just be limited by other reasons, for example, by a lack of oxygen and/or lack of glucose. The cell growth could develop more favorably in practical applications than shown in Figure 11, since vascularization could lift this limitation. An hypothetic lack of oxygen and or glucose, in turn, could occur when a high number of cells is seeded: they rapidly fill the pores reducing the channel space for oxygen and/or glucose to reach the center of the scaffold. If this hypothesis is correct, the cell number needs to be modulated depending on pore size and scaffold dimension.

Viability tests we did also demonstrated that resazurin assay was not toxic for hMSCs on biocorals after 2 h incubation time allowing a invasive monitoring over 21 days and Biocoral chemical composition did not alter the fluorescence of resazurin. However, resazurin has been found cytotoxic and induces an antiproliferative effect in some cancer cells after incubation times between 6 and 48 h, as found by Erikstein *et al*. [42] and also reported by Liss *et al*. [24]. In our experiments incubation time is much shorter (2 h) and the cell type we tested (hMSCs), did not show such drawback effects seen in cancer cells, even when cultured in traditional 2D cell cultures. Apoptosis test could be done to more deeply assess the resazurin toxicity on hMSCs.

Perrot *et al*. in 2003 [31] measured the emission spectra of a resazurin solution 40 μM and a resorufin solution 40 μM, both solutions prepared in methanol. They excited at 570 nm and obtained a maximum emission at 590 for resorufin and two different peaks for resazurin with a maximum at 630 nm. However, the fluorescence intensity of resazurin was negligible. These data have been confirmed by our data, even if the two different peaks found in nonmetabolized resazurin by Perrot, in our experiments were found in resazurin metabolized by low number of cells. This confirms our hypothesis that the solution metabolized by cells is a mixture of resazurin and resorufin at relative concentrations that change with the number of cells (or with the incubation time). We expect to find, in our future experiments, that different spectra can be correlated to different cell number.

We also measured the fluorescence stability of samples which are collected and stored until the measurement is performed. Fluorescence intensity measurements were performed at different incubation times soon after removing cells from the incubator. Then, the metabolized resazurin was withdrawn from the cells and was stored 24 h at room temperature and fluorescence was measured. Incubator maintains the temperature around 37.0°C and the pH in the range of 7.0–7.4 though the 5% CO_2_ insufflation. This pH range is optimal pH for hMSCs *in vitro* growth and is also optimal for resazurin assay as reported by Rampersad [32]. At room temperature (in the specific case 23°C) the fluorescence intensity of the same sample of metabolized resazurin decreased, as shown in Figure 9, and also pH decrease (data not shown). Fluorescence must be read as soon as the incubation time finishes to avoid fluorescence changes probably due to temperature and pH decrease. Another important factor is that fluorescence should come only from the metabolized resazurin (i.e., resorufin) and no interference should come from the growth medium in which cells are cultured and treated with resazurin. However, the medium for cell culture often contains phenol red as pH indicator which gives a red/yellow color to the medium depending on the pH and could interfere with fluorescence measurements. Also, the cell culture medium is normally added with FBS to provide the cells with the appropriate amount of growth factors, hormones and detoxifying substances for the survival and proliferation of cells in culture. FBS is known to be auto-fluorescent [43]. With the final aim to optimize the measurement condition in terms of culture media, we tested the possible auto-fluorescence of different cell culture media that were found negligible. For this reason we decided to use the same cell culture medium for culturing and for resazurin assay in order to not perturb the conditions in which the cells are growing.

Results showed that the incubation time needs to be adjusted depending on the cell number you want to discriminate, so it is cell number-dependent, as also suggested by Rampersad [32] for 2D cell cultures: low number of cells shows a slower proliferation rate and needs incubation times shorter than high number of cells. In a 3D cell culture, the scaffold dimension also plays an important decision making role. For our purposes it was necessary to find the optimal incubation time able to give good results for both low and high number of cells, relatively to the scaffold dimensions. The incubation time of 2 h was the best on both the 3D and 2D allowing a reliable detection of cells on a wide range of cell numbers for application in regenerative medicine on biocorals 5 × 9 × 11 mm. This incubation time avoids the dilution of metabolized resazurin, necessary when off-scale occurs. Dilution is not advisable because the linearity is lost or a calibration curve *ad hoc* for dilutions needs to be realized.

The manufacturer [23] suggests to stop the resazurin reaction by adding sodium dodecyl sulfate, which dilutes the sample, and, if necessary, to read the fluorescence after storing the sample in the dark at room temperature until 24 h. We showed, as above stated, that resazurin fluorescence changes over time with the temperature, even if far from the cells and this change is different with three different samples. In addition, the dilution of the sample not always maintains the linear correlation we expect. Results we found on the dilution and on the storage at room temperature suggest that it is highly recommended to measure the fluorescence as soon as taking the cells out from the incubator and to avoid dilutions.

## Conclusion

The method here proposed and characterized for 3D cell proliferation monitoring in a noninvasive way, showed that Biocoral allowed hMSCs seeding and colonization confirming what demonstrated by Zhu *et al*. [19]; the method did not interfere with cellular viability and proliferation was followed over long-time period (21 days) on 21 different scaffolds, with a good repeatability and reproducibility.

This is strongly reinforced if considering the intrinsic variability of this complex system of 3D cell cultures: the Biocoral has a not reproducible internal structure which is formed by “naturally made” interconnected pores and channels. The proliferation curves we produced through the proposed method, can be a powerful tool for clinicians to make hypothesis and take important decisions on the cell dose in order to have the desired effect on patients, by translating the *in vitro* clues into clinical applications. As an example, it is possible to set the cell number to be seeded in order to modulate the proliferation rate and reach the desired cell density within the scaffold over time. The method allows multiplexing assays, for example, the cell number measurement could be followed by a viability/apoptosis assay and finally by a gene expression analysis with the aim to correlate the number of viable cells with the cell differentiation state. This could lead, for instance, to the modulation of the number of cells to stimulate the differentiation within a certain period of time and so on. In this way, by working backwards the cell dose can be found as the number of cells needed at the seeding to develop the correct tissue after transplantation.

Concluding, the method could be a good support for the cell dose determination in regenerative medicine applications and in general for cell–scaffold interaction studies.

## Future perspective

This study represents a first approach toward the development of a noninvasive, reproducible method for 3D cell proliferation measurement. The cell dose determination is a critical point in regenerative medicine and tissue engineering. However, there are few noninvasive methods investigating the number of cells within a highly porous scaffold and the combination of more methods, preferably noninvasive, is still needed. The method here proposed will be improved and supported by more data to evaluate the reproducibility on a larger number of scaffold samples and on dynamic cultures, which have demonstrated better results on cell proliferation than static cultures. Also, experiments on the oxygen shortage and supply will be performed in a future study. In addition, analysis by micro-CT will be taken into consideration to combine morphology analysis with those on cell dose coming from the resazurin-based method.

**Executive summary**

**Aim**

The authors wished to demonstrate the use and the characterization of the resazurin-based metabolic cell proliferation assay on 3D cell cultures in highly porous scaffold in order to evaluate the cell dose for regenerative medicine applications.

**Results**

Using optical microscopy and scanning electron microscopy it is shown the pore dimension distribution of the Biocoral scaffold and the scaffold internal structure, respectively.
Using fluorescence intensity spectrophotometer, it is shown that the two forms of resazurin, metabolized and not metabolized by cells, have two different spectra.
An extensive characterization of the resazurin-based method is performed to identify the better experimental conditions in terms of resazurin concentration, incubation time and cell growth medium composition.
A characterization of the fluorescence multiplate reader showed that the instrumental repeatability is 0.5% and instrumental reproducibility is 1%.
The method based on resazurin metabolism is shown linear in a wide range of cell number between 5 × 10^3^ and 4.0 × 10^5^ human mesenchymal stem cells. The correlation coefficient was 0.97. Sensitivity (5000 cells), repeatability (0.0 and 5.2%) and reproducibility (10.0–20.5%) are evaluated for initial cell number between 2.5 × 10^3^ and 3.0 × 10^5^ cells/Biocoral.
The uncertainty of the method is evaluated taking into account repeatability, reproducibility and the major factor of uncertainty and is calculated as less than 6%, acceptable for regenerative medicine field.
The method is not toxic for human mesenchymal stem cells as demonstrated by Calcein AM vital staining and fluorescence microscopy.
Proliferation curves are created at different initial cell number and data are compared with traditional bidimensional cell cultures. A total number of 21 biocorals are analyzed over 21 days.
Data on cell dose show that the initial cell number strongly influences the cell proliferation trend over time and the pore dimension is probably the most influencing factor on the cell proliferation rate.

**Conclusion**

We proposed a robust protocol for cell number assessment in Biocoral scaffolds allowing reproducible *in vitro* analysis of the cell dose in 3D cell cultures with an associated uncertainty. This allows to make several deductions on the cell behavior within scaffolds and could be a useful tool to determine the cell dose for clinical applications.
